# Combinatorial genetic manipulation of Cx50, PI3K and PTEN alters postnatal mouse lens growth and homeostasis

**DOI:** 10.3389/fopht.2025.1502836

**Published:** 2025-02-19

**Authors:** Caterina Sellitto, Thomas W. White

**Affiliations:** Department of Physiology and Biophysics, Stony Brook University School of Medicine, Stony Brook, NY, United States

**Keywords:** lens, growth, cataract, connexin, PTEN, PI3K, mouse model

## Abstract

**Introduction:**

Phosphoinositide 3-kinase (PI3K), Phosphatase and tensin homolog (PTEN) and connexin50 (Cx50) have individually been shown to play critical roles in the growth, development and maintenance of the lens and to functionally interact *in vitro*. To elucidate how gap junctional coupling mediated by Cx50 and intracellular signaling mediated by PI3K and PTEN synergistically interact to regulate lens homeostasis *in vivo*, we generated and characterized double knockout animal models lacking the p110α subunit of PI3K and Cx50, or PTEN and Cx50.

**Methods:**

We interbred lens specific p110α and PTEN conditional knockout animals with Cx50 deficient mice to generate double knockouts. Animals and eyes were weighed, lenses were dissected, photographed, measured, fixed and sectioned for histological analysis. Lens epithelial cell proliferation was determined using 5-ethynyl-2’-deoxyuridine (EdU) labeling.

**Results:**

Double knockout of p110α and Cx50 led to a significant reduction in lens and eye size, and a high rate of lens rupture. The individual cell proliferation defects of the Cx50 and p110α single knockout lenses both persisted in the double KO. Double deletion of Cx50 and PTEN produced severe lens defects, including cataract, aberrant cell migration, altered cell proliferation, vacuole formation and lens rupture.

**Conclusion:**

The severe phenotypes in p110α/Cx50 and PTEN/Cx50 double deficient lenses suggest that PI3K, PTEN and Cx50 participate in both distinct and common regulatory pathways that are necessary to maintain normal lens growth and homeostasis.

## 1 Introduction

Multicellular organisms utilize several mechanisms to provide the intercellular communication needed between cells to achieve the normal growth, differentiation and maintenance of organs. These mechanisms can include communication being directly mediated by the connexin channels present in gap junctions (1–3), or driven by extracellular growth factors binding to receptors and activating intracellular signal transduction cascades (4, 5). Both signal transduction pathways and connexin mediated communication have been shown to play critical roles in the development of the eye lens by numerous laboratories (5–23), however, less is known about the potential interplay between them (24).

Gap junction channels facilitate the direct transport of ions, metabolites, and small signaling molecules between cells of the lens (25). Gap junctions are comprised of hexameric oligomers of connexin proteins (26). When two of these complexes from neighboring cells dock, they form a channel that directly connects the cytoplasm of two cells (27). Three connexins are present in the lens with different patterns of expression: Connexin43 (Cx43) is present in the lens epithelium (28), Connexin46 (Cx46) is expressed in the lens fiber cells (29), and Connexin50 (Cx50) is highly abundant in both cell types (13, 30, 31). Knockout of Cx43 in the lens had no detectable phenotype, while loss of Cx46 resulted in cataract (32–34). By contrast, deletion of Cx50 significantly reduced postnatal lens cell proliferation, particularly in the central epithelium, resulting in deficient lens growth, micropthalmia and cataract (13, 15, 17, 35).

Phosphoinositide 3-kinases (PI3Ks) act downstream of cell receptors to phosphorylate the 3′-hydroxyl group of phosphatidylinositol- (4, 5)P_2_. This generates phosphatidylinositol- (3–5)P_3_ (PIP_3_), which then activates signaling pathways to regulate cell growth, proliferation, and survival (36). PI3Ks are separated into different classes depending upon sequence homology and substrate specificity (37). Class IA enzymes are heterodimers composed of 110kD catalytic and 85kD regulatory subunits. The p110α catalytic subunit is widely expressed, and responds to input from receptor tyrosine kinases (38), generating PIP_3_, whose most prominent biological function is the activation of the AKT signaling pathway (39–42). PI3K signaling is antagonized by phosphatase and tensin homolog (PTEN), a ubiquitously expressed lipid phosphatase that dephosphorylates PIP_3_ (43, 44). Regulatory control mediated by the interplay between PI3K and PTEN governs numerous cellular processes in many organs, including the lens (45–47).

The lens contains a monolayer of epithelial cells covering the anterior surface, with fiber cells derived from epithelial precursors filling its core (48). Epithelial proliferation drives lens growth, which predominantly occurs near the equator in the germinative zone (49–51). Lens growth and development are regulated by growth factor signaling, such as that provided by the fibroblast growth factors (FGFs) and fibroblast growth factor receptors (FGFRs) that control lens induction, epithelial cell proliferation and fiber differentiation (5, 18, 52). FGFRs are receptor tyrosine kinases that stimulate the mitogen-activated protein kinase (MAPK, or Ras-Raf-Mek-ErK) or PI3K-AKT intracellular signaling pathways (45, 53, 54). Components of the MAPK pathway have been extensively studied in the lens by genetic dissection using transgenic mice (9, 10, 55–59).

Elucidation of the role(s) played by components of the PI3K/PTEN branch of the intracellular signaling pathway has also been explored using genetically engineered mice. Lens specific deletion of PTEN induced elevated levels of phosphorylated AKT, with distinct consequences depending upon the developmental timing of deletion (6, 60). Deletion at the lens placode stage rescued a cell death phenotype caused by knockout of FGFR2 (6), whereas deletion at the lens vesicle stage inhibited Na^+^/K^+^-ATPase activity, leading to lens rupture and cataract (60). Lens specific conditional knockouts of the p110α and p110β catalytic subunits of PI3K alone, or in combination, have also been generated (16). Deletion of p110α significantly reduced eye and lens growth due to altered spatial organization, and a reduced magnitude of lens epithelial cell proliferation on postnatal day 0 (P0). Deletion of p110β did not induce a detectable phenotype, and mice with double knockout of p110α and p110β had the same lens phenotype as p110α single knockout animals (16).

We have previously shown that both Cx50 and PI3K individually regulate postnatal lens cell proliferation and growth *in vivo* (15, 16, 35). We have also demonstrated that PI3K can specifically modulate the functional activity of Cx50 *in vitro* (61). We have further established that PI3K/PTEN signaling critically regulates the activity of a broad network of lens ion channels and transporters, including Cx50 (60, 62–64). We do not currently know if Cx50, PI3K and PTEN operate independently, or in a common pathway, in the regulation of lens cell proliferation. We also do not understand the mechanism(s) whereby PI3K/PTEN signaling leads to Cx50 dependent changes in lens differentiation and homeostasis. To address how Cx50 and PI3K/PTEN signaling work together in the lens to maintain clarity, preserve integrity and regulate postnatal mitosis, we have examined the *in vivo* functional interactions between PI3K, PTEN, and Cx50 using double knockout mouse models.

## 2 Materials and methods

### 2.1 Generation of double knockout animals

The Stony Brook University Institutional Animal Care and Use Committee approved all animal experimentation. Mice with a global knockout of Cx50 (17) and lens-specific conditional knockouts of the p110α catalytic subunit of PI3K (16), or PTEN (60), were mated to generate double knockout animals (p110/Cx50α dKO, or PTEN/Cx50 dKO). Briefly, Cx50 KO and p110α floxed (65) mice were interbred to homozygosity. Cx50 KO and PTEN floxed mice (66) were also interbred to homozygosity. MLR10-Cre mice, whose Cryaa driven Cre expression is limited to the lens epithelium and fibers (67), were interbred with both strains to heterozygosity, maintaining the homozygous floxed p110α and PTEN alleles, in addition to the homozygous Cx50 KO alleles. Animal genotypes were confirmed by PCR of DNA from tail biopsies as previously described (16, 17, 60). Due to the mixed genetic background that resulted from the interbreeding of the different strains of original mice, littermate controls (Cx50 KO Cre negative) were used for all experiments. All animals used for breeding had homozygous Cx50 KO and homozygous floxed alleles for either PTEN or p110α, while one member of the breeding pair carried a heterozygous copy of the MLR10-cre transgene. The resulting litters contained half of the animals with no Cre (Cx50 KO controls) and half with a double knockout of p110α/Cx50, or a double knockout of PTEN/Cx50 (dKOs).

### 2.2 Lens photography and growth measurement

Littermate mice between birth and 24 weeks of age were euthanized by CO_2_ asphyxia and weighed. Eyes were dissected, weighed, and transferred to 37°C Tyrode solution on a warmed stage. Lenses were removed, transferred to 35mm glass bottom culture dishes and photographed with a SZX16 dissecting microscope attached to a digital camera (Olympus, Waltham, MA). Diameters of lenses were measured from the images, and lens volume was calculated assuming a spherical shape. The incidences of the presence of blood vessels associated with the lens and lens rupture were also recorded (16, 17, 60).

### 2.3 5-ethynyl-2’-deoxyuridine labeling

Postnatal day 0 (P0) or postnatal day 2 (P2) mice were subcutaneously injected with 50 µg/gm EdU (Click-iT, Thermo Fisher Scientific, Waltham, MA) and then returned to their mothers for 2 hours. Subsequently, lenses were removed and fixed in a 4% formaldehyde in PBS for 1 hour at room temperature (~22°C) and photographed for measurement. Permeabilization and Click-iT staining were performed as described in the manufacturer’s instructions and previously published protocols (16, 68, 69). Z-stacks of fluorescent images were acquired on an Axiovert 200M microscope (Zeiss, Thornwood, NY) and processed using ImageJ. For line-scan analysis, the flattened fluorescent image of EdU staining was manually thresholded, and the plot profile function in ImageJ was used to measure fluorescent intensity across the entire lens diameter as described previously (16, 68). For central epithelial cell counts, a circular region with a diameter equal to the lens radius was drawn over the central region, fluorescent values were thresholded using the color threshold fuction in ImageJ, and EdU stained cells were quantified using the analyze particles function in ImageJ. The number of labeled cells was divided by the circular area to calculate the density of labeled cells.

### 2.4 Histological staining

Eyes from postnatal day 2 mice were dissected and fixed in 4% formaldehyde in phosphate-buffered saline (PBS) overnight at room temperature. Eyes were rinsed with PBS, dehydrated through increasing ethanol concentrations (50, 70, 70, 80, 95, 100%), and then embedded in paraffin. 2.5 µm sections were cut using a diamond knife, mounted on glass slides and deparaffinized. Slides were stained with hematoxylin-eosin as previously described (16, 60), and histological sections were observed on a BX51 microscope and photographed with a DP72 digital camera (Olympus).

### 2.5 Statistical analysis

Data were plotted as the mean ± SD, or SEM, as described in each figure. Statistical significance was determined using one-way ANOVA in the Origin software program (OriginLab Corporation, Northampton, MA) with Tukey’s *post hoc* test. P values less than 0.05 were considered significant.

## 3 Results

### 3.1 Double knockout of p110α/Cx50 or PTEN/Cx50 altered lens size, clarity and integrity

**Figure 1:**
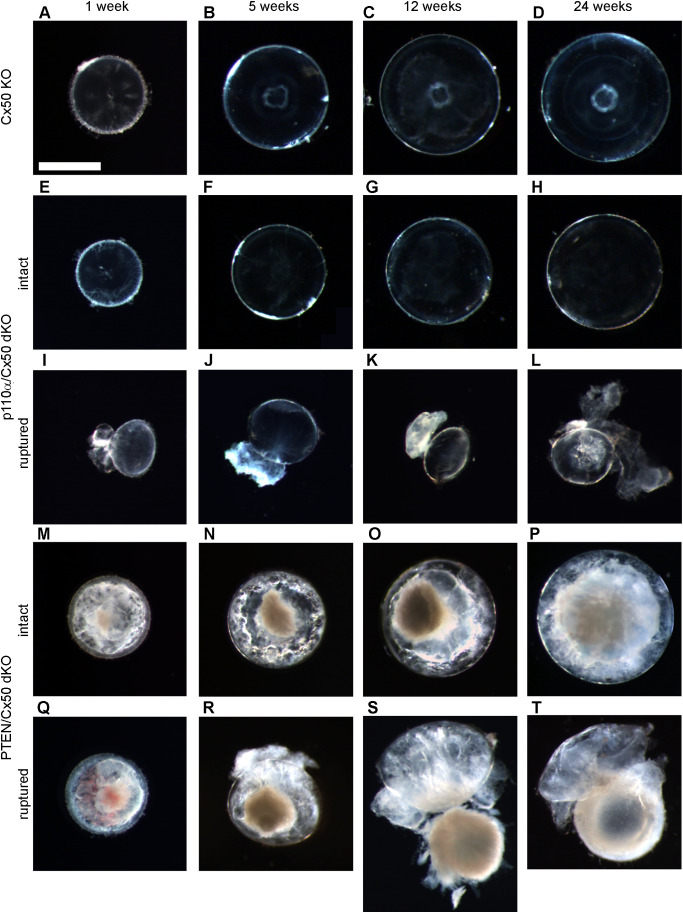
p110α/Cx50 dKO and PTEN/Cx50 dKO mice show altered lens size, clarity and integrity. Lenses were dissected and photographed at 1, 5, 12, and 24 weeks of age. Cx50 KO lenses **(A–D)** were intact at all ages studied and contained a mild nuclear cataract. p110α/Cx50 dKO lenses that did not rupture **(E–H)** were smaller than single Cx50 KOs, and lacked the mild nuclear cataract. Most p110α/Cx50 double KO lenses **(I–L)** displayed a partial posterior rupture, leaving a transparent anterior lens fragment attached to posterior lens debris. PTEN/Cx50 dKO lenses displayed a more severe phenotype than single Cx50 KO or PTEN KO lenses. Intact lenses **(M–P)** displayed a dense nuclear cataract and abundant cortical vacuoles. The incidence of total lens rupture increased with age **(Q–T)**. Bar = 1mm, all panels are at the same magnification.

Cx50 KO mice (17) containing homozygous floxed p110α, or PTEN alleles (65, 66) were interbred with MLR10-Cre transgenic mice (67) to obtain double knockout animals. All comparisons in this manuscript are made between animals lacking Cx50. As described in the introduction, loss of Cx50 reduced postnatal lens cell proliferation, causing deficient lens growth, micropthalmia and cataract (13, 15, 17, 35). Please see **Table 1**, and the original knockout papers (16, 17, 60) for a comparison of the single KO mouse phenotypes with wild-type lenses. Lenses isolated from homozygous floxed p110α/Cx50, or PTEN/Cx50 animals lacking the MLR10-Cre transgene (i.e. Cx50 KO) remained intact at all ages examined (**Figures 1A–D**), with a mild nuclear cataract as previously described (70). By contrast, lenses dissected between 1 and 24 weeks of age from homozygous floxed p110α/Cx50 animals expressing the MLR10-Cre transgene (i.e. p110α/Cx50 dKO) showed a distinct dual pathology. Intact lenses formed the first group, which were noticeably smaller than their Cx50 KO littermates, and no longer displayed the mild nuclear cataract phenotype of the Cx50 KO (**Figures 1E–H**). The second group of p110α/Cx50 dKO lenses displayed a partial posterior rupture, leaving a small transparent anterior lens fragment attached to posterior lens debris (**Figures 1I–L**). Lenses dissected between 1 and 24 weeks of age from homozygous floxed PTEN/Cx50 mice expressing the MLR10-Cre transgene (i.e. PTEN/Cx50 dKO) showed a progressive pathology that also included lens rupture. A dense central cataract, the presence of vacuoles, and progressively worsening cortical opacities, characterized the PTEN/Cx50 dKO lenses that remained intact (**Figures 1M–P**). Lens rupture was not detected at 1 week of age for PTEN/Cx50 dKO lenses, however from 5 weeks of age onward, ruptured lenses were observed with an increasing frequency leaving behind a dense nuclear cataract attached to cortical fragments (**Figures 1Q–T**). Thus, lens size, clarity, and integrity were all differentially impacted by the double deletion of Cx50 with p110α, or PTEN. p110α/Cx50 dKO lenses were prone to lens rupture, however, intact p110α/Cx50 dKO lenses were much smaller than Cx50KOs, and had lost the central cataract. PTEN/Cx50 dKO lenses had more severe cataracts that either Cx50, or PTEN single KOs, and displayed frequent lens rupture.

**Table 1:** Summary of phenotypic differences between single and double knockout animals.

knockout(s)	eye mass	lens volume	epithelial cell proliferation	lens rupture	reference(s)
Cx50	reduced 32%	reduced 44%	reduced on P2 in central zone	none	(15, 17)
p110α	reduced 22%	reduced 27%	reduced on P0 in germinative zone	none	(16, 68)
PTEN	increased 19%	increased 29%	no change	> 50% at 24 weeks	(60, 68)
p110α/PTEN	normal	increased 22%	no change	> 50% at 12 weeks	(68)
p110α/Cx50	reduced 55%	reduced 62%	reduced on P0 in germinative zone	> 50% at 1 week	present work
PTEN/Cx50	reduced 27%	reduced 40%	reduced on P2 in central zone	> 50% at 24 weeks	present work

Comparisons of body mass, organ mass, organ volume, cell proliferation and organ rupture are all made to aggregate wild-type data from previous publications (15–17, 60, 68).

### 3.2 Double deletion of p110α/Cx50 resulted in reduced eye and lens growth

**Figure 2:**
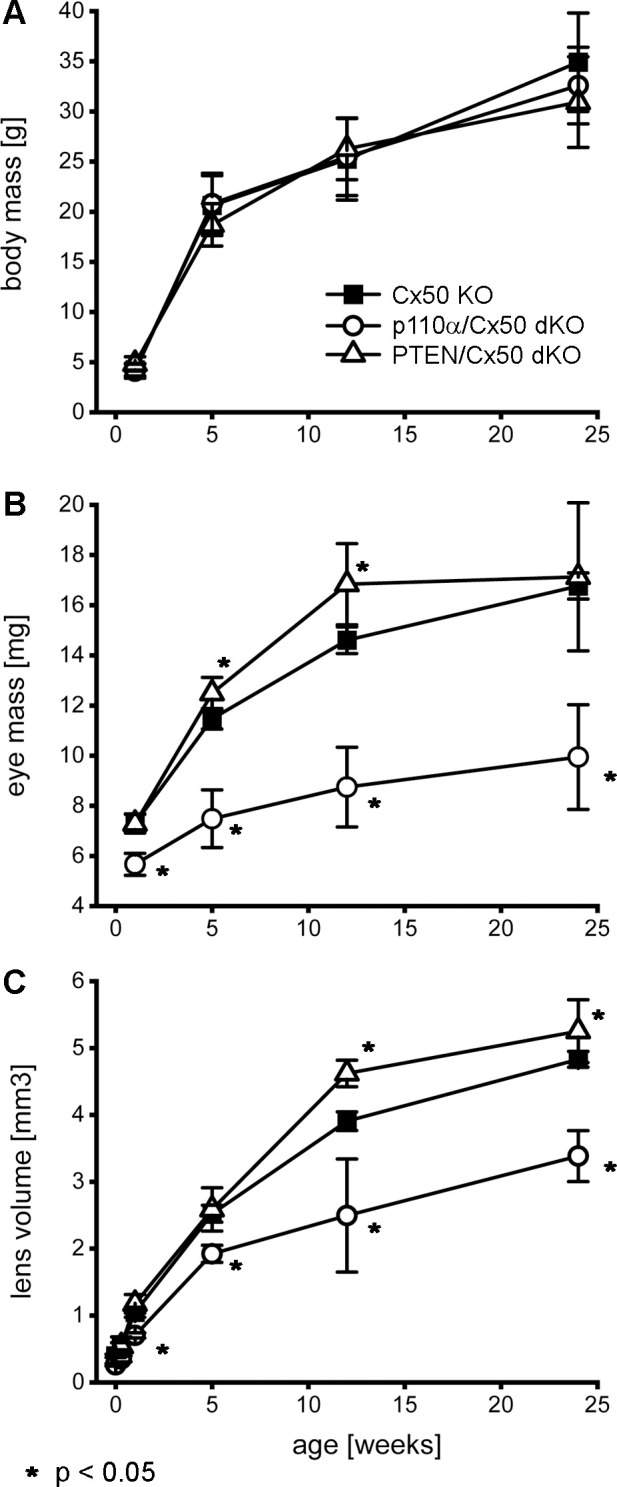
Double knockout of p110α/Cx50 reduced eye and lens growth. Changes in animal mass, eye mass and lens volume were quantified between P0 and 24 weeks of age. Body mass **(A)** for Cx50 KO, p110α/Cx50 dKO, and PTEN/Cx50 dKO animals was similar at all ages (n = 8-27 animals/genotype/age). Eye mass **(B)** in the p110α/Cx50 dKO was significantly reduced at all ages (23-40% smaller, p < 0.05, one-way ANOVA, n = 16-42 eyes/genotype/age) compared to either the Cx50 KO or PTEN/Cx50 dKO mice. Between 5 and 12 weeks, eyes from PTEN/Cx50 dKO animals were slightly larger than Cx50 KO (8-15% increase, p < 0.05). Lens volume **(C)** in the p110α/Cx50 dKO was significantly reduced (up to 37% smaller, p < 0.05, n = 6-36 lenses/genotype/age). Lens volume in the PTEN/Cx50 dKO animals were increased between 12 and 24 weeks of age (8-18% larger, p < 0.05). Data are plotted as mean ± SD. Asterisks indicate significantly different values compared to the Cx50 KO.

Previous studies reported that individual knockouts of either Cx50, or p110α, resulted in the lens volume being reduced by 44% and 27%, respectively with a corresponding decrease in eye mass (13, 16, 17). By contrast, single knockout of PTEN resulted in a small increase in lens volume and eye mass (60). To determine the effect of double knockout of Cx50 and p110α or PTEN, changes in animal mass, eye mass and lens volume were quantified over time by weighing mice, weighing eyes, and photographing lenses between postnatal day 0 (P0) and 24 weeks of age in Cx50 KO, p110α/Cx50 dKO and PTEN/Cx50 dKO animals. Lens diameters were measured, and then used to calculate lens volumes as previously described (16, 17, 60, 68). There were no significant differences in body mass between Cx50 KO, p110α/Cx50 dKO and PTEN/Cx50 dKO animals between birth and 24 weeks of age (**Figure 2A**). By contrast, eye mass in the p110α/Cx50 dKO was significantly reduced (p < 0.05, one-way ANOVA) compared to Cx50 KO and PTEN/Cx50 dKO mice (**Figure 2B**). At 1 week old, the mass of p110α/Cx50 dKO eyes was reduced 23% and by 12 weeks of age, the eyes were 40% smaller than Cx50 KO mice. By contrast, eyes from PTEN/Cx50 dKO animals were 8-15% larger than Cx50 KO littermates at 5 and 12 weeks of age (p < 0.05). The eye mass data includes all eye samples, regardless of lens rupture status. Similar to eye mass, lens volume in the p110α/Cx50 dKO animals was significantly reduced (p < 0.05) compared to either the Cx50 KO or PTEN/Cx50 dKO (**Figure 2C**). At P0, the volume of p110α/Cx50 dKO lenses was reduced 35% and at 12 weeks of age the lenses were 37% smaller than those of Cx50 KOs. The lenses from PTEN/Cx50 dKO animals were 8-18% larger than Cx50 KO littermates between 12 and 24 weeks of age (p < 0.05). The lens volume data was calculated from lenses that had not ruptured. These data show that the eye and lens growth deficiencies induced by individual knockout of Cx50 or p110α were compounded in the p110α/Cx50 dKO. The modest effect of single PTEN KO on lens and eye size (60) was not notably altered in the PTEN/Cx50 dKO animals.

### 3.3 Double knockout of Cx50 and p110α or PTEN increased lens rupture

**Figure 3:**
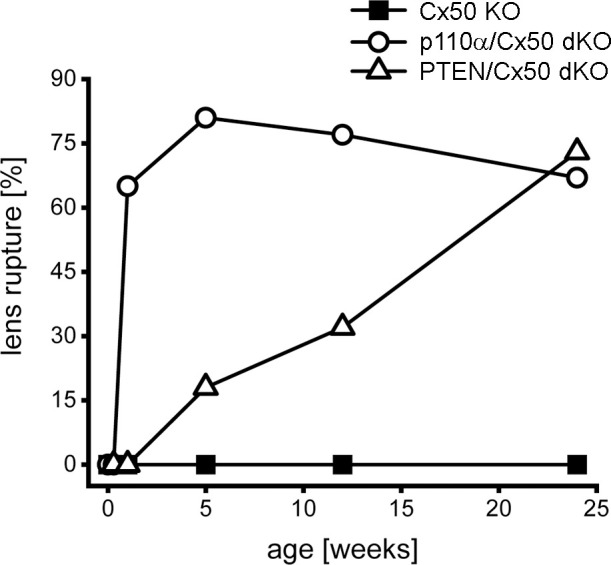
Lens rupture increased in p110α/Cx50 and PTEN/Cx50 dKO mice. The incidence of lens rupture was plotted against age between P0 and 24 weeks. No lens rupture was observed in Cx50 KO animals. Lenses from p110α/Cx50 dKO mice were intact up until P2, but then displayed high rates (65-81%) of rupture after 1 week. PTEN/Cx50 dKO lenses displayed an increasing propensity to rupture with age, than was accelerated compared to single PTEN KO mice (60). n = 16-42 lenses/genotype/age.

Previous studies have shown that single Cx50, or p110α, KO lenses never displayed lens rupture (16, 17, 68). By contrast, single PTEN KO lenses displayed an increasing propensity to rupture with age (60). To quantify changes in lens integrity over time, dissected lenses from Cx50 KO, p110α/Cx50 dKO and PTEN/Cx50 dKO animals between birth and 24 weeks were scored for the incidence of lens rupture (**Figure 3**). Reduced viability or anophthalmia were never observed in either dKO strain. Lenses from p110α/Cx50 dKO mice were intact between P0 and P2, but displayed 65% to 81% rupture between 1 week and 24 weeks of age. Lenses from PTEN/Cx50 dKO mice were intact between P0 and 1 week of age. By 5 weeks of age, PTEN/Cx50 dKO animals had an 18% incidence of lens rupture, which increased to 32% at 12 weeks, and 73% at 24 weeks of age. These data show that loss of either p110α or PTEN from Cx50 KO lenses produced a loss of lens integrity and high incidences of lens rupture. However, the kinetics of lens ruptured differed dramatically between the p110α/Cx50 dKO and PTEN/Cx50 dKO mice.

### 3.4 Double deletion of PTEN/Cx50 alters the association of blood vessels with the early postnatal lens

**Figure 4:**
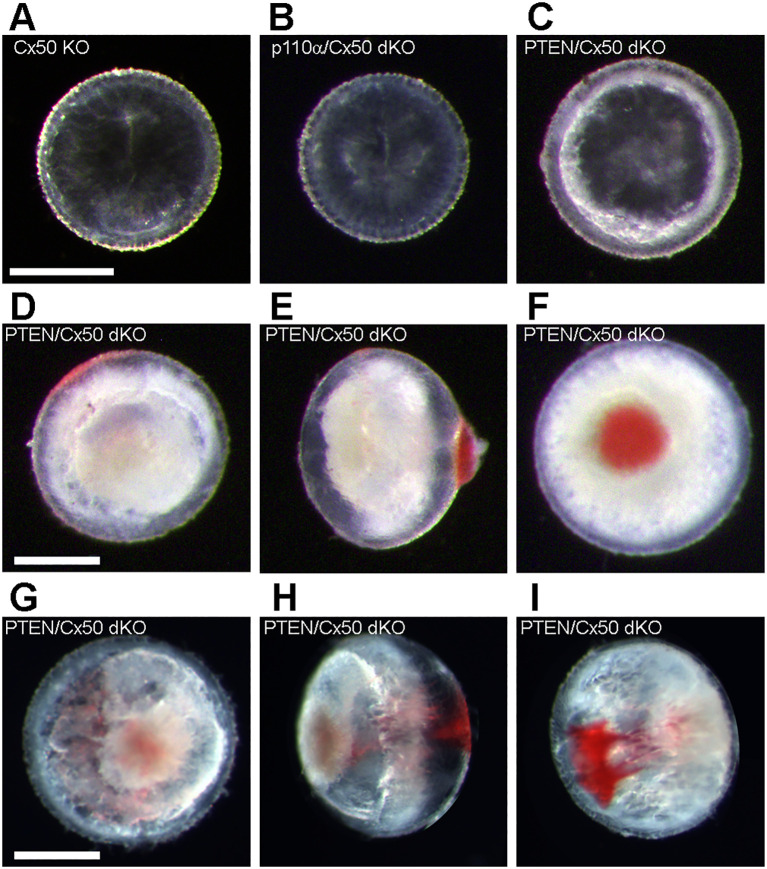
Postnatal PTEN/Cx50 dKO lenses show altered interaction with blood vessels. The tunica vasculosa lentis provides a blood supply to the developing lens during the first postnatal week (71–74). All P2 lenses from Cx50 KO **(A)** and p110α/Cx50 dKO **(B)** could be dissected away from the blood vessels of the tunica vasculosa. Most lenses (71%, n = 14) from PTEN/Cx50 dKO animals could also be separated from the tunica vasculosa on P2 **(C)**. However, 29% of P2 PTEN/Cx50 dKO lenses could not be separated from highly adherent/penetrant blood vessels (**D**, anterior view, **E** side view, **F** posterior view). On P7, 13% (n = 16) of PTEN/Cx50 dKO lenses were associated with tunica vasculosa blood vessels (**G**, anterior view, **H** side view, **I** posterior view). Bars = 0.5 mm.

In the early mouse eye, the hyaloid artery grows out from the optic nerve head ensheathing the lens as the tunica vasculosa lentis (71, 72). This structure persists during the first postnatal week before regressing prior to eye opening on P14 (73, 74). In PTEN/Cx50 dKO mice, this process appeared to be compromised in a subset of lenses dissected between P2 and P7. All of the postnatal lenses from Cx50 KO and p110α/Cx50 dKO, and the majority of lenses from PTEN/Cx50 dKO animals could be easily dissected away from the tunica vasculosa on P2 (**Figures 4A–C**). However, 29% (n = 14) of the P2 lenses (**Figures 4D–F**) and 13% (n = 16) of the P7 lenses (**Figures 4G–I**) from PTEN/Cx50 dKO could not be separated from highly adherent/penetrant blood vessels that presumably originated from the tunica vasculosa lentis. This phenomenon was not previously observed in wild-type, single Cx50 KO, or single PTEN KO eyes (13, 17, 60).

### 3.5 The P0 proliferation defect present in single p110α KO lenses persisted in p110α/Cx50 dKO mice

**Figure 5:**
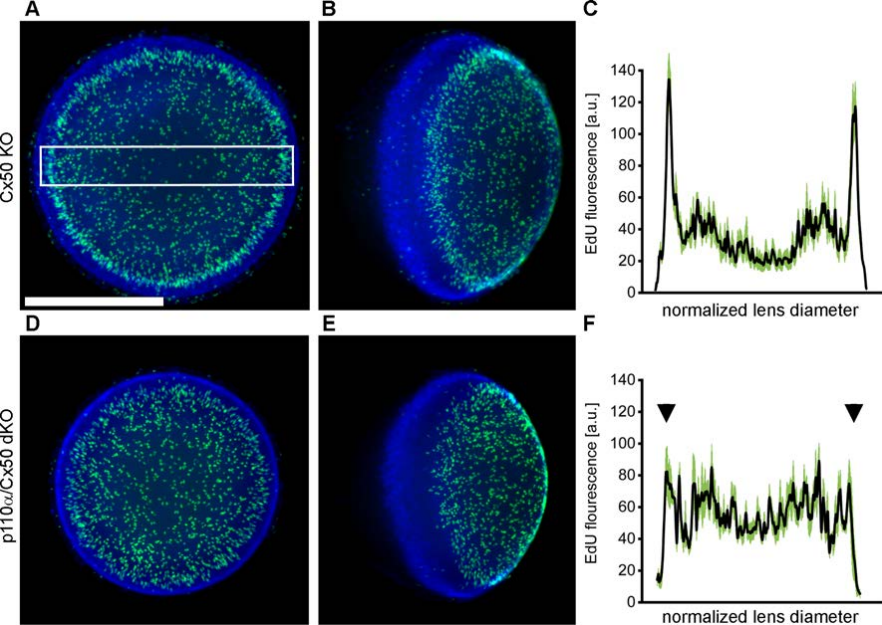
P0 p110α/Cx50 dKO lenses showed a large reduction in cell division in the equatorial germinative zone of the lens epithelium. Lenses from Cx50 KO **(A, B)** and p110α/Cx50 dKO **(D, E)** animals were labeled with EdU (green **A, B, D, E**) and Hoechst (blue **A, B, D, E**) on P0. Z-stack images were created looking onto the epithelial surface **(A, D)**, or onto the lens edge **(B, E)**. Plots of mean values of line scans (black lines, ± the SEM green bars) of EdU fluorescence taken across the lens diameter **(C, F)** showed that the p110α/dKO lenses **(F)** had reduced peak proliferation in the equatorial germinative zone (black arrowheads) compared to Cx50 KO lenses **(C)**. n = 4-5 lenses per genotype. White boxed area in A depicts a representative scan region. Bar = 0.5 mm.

The pattern of mitosis in p110α single KO lenses was altered on P0, with a large reduction in cell division in the equatorial germinative zone of the lens epithelium (16, 68). To examine postnatal mitosis in p110α/Cx50 mice, lenses from Cx50 KO and p110α/Cx50 dKO mice were labeled with EdU on P0. Cx50 KO lenses showed strong EdU labeling, with the highest level of fluorescence in the germinative zone near the lens equator (**Figures 5A, B**). By contrast, p110α/Cx50 dKO lenses lacked the ring of increased labeling near the equatorial germinative zone, and showed a more uniform pattern of EdU incorporation across the lens epithelium (**Figures 5D, E**). Line scans of the mean fluorescent intensity taken across the lens diameter (black lines, ± the SEM green bars) confirmed this change in mitotic pattern between EdU labeled images from P0 Cx50 KO (**Figure 5C**) and p110α/Cx50 dKO lenses (**Figure 5F**, arrowheads). All P0 Cx50 KO lenses displayed large peaks of EdU fluorescent intensity in the equatorial germinative zone (n = 5). By contrast, all P0 p110α/Cx50 dKO lenses lacked the equatorial peaks, and had maximum mean values of fluorescence that ranged from 35% to 39% lower than Cx50 KO lenses (n =4). These data show that further deletion of Cx50 from p110α knockout lenses did not restore normal P0 epithelial cell proliferation.

### 3.6 PTEN/Cx50 dKO lenses did not rescue central epithelial proliferation and displayed altered distribution of nuclei on P2

**Figure 6:**
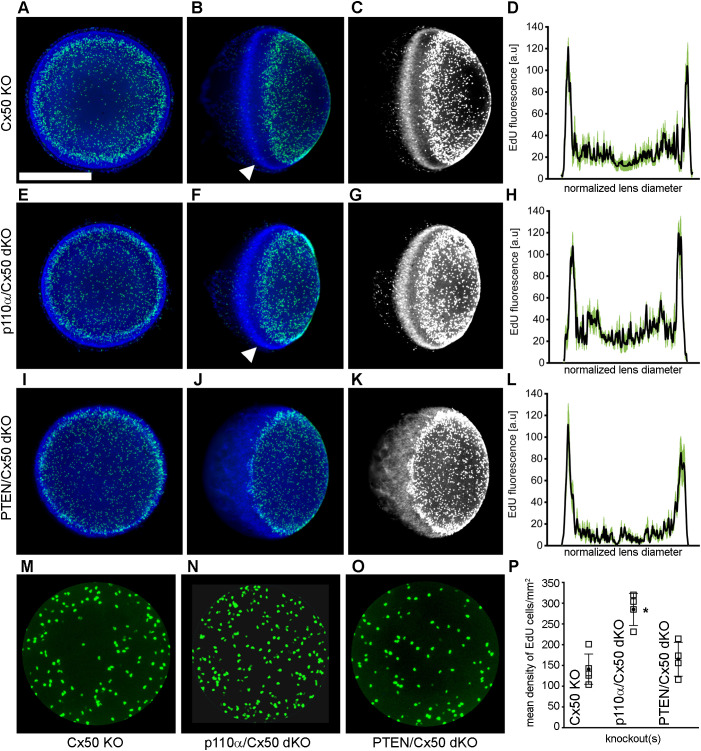
PTEN/Cx50 dKO lenses did not rescue central epithelial proliferation and displayed altered distribution of nuclei on P2. Lenses from Cx50 KO **(A–C)**, p110α/Cx50 dKO **(E–G)** and PTEN/Cx50 dKO **(I–K)** animals were labeled with EdU (green **A, B, E, F, I, J**) and Hoechst (blue **A, B, E, F, I, J**) on P2. High contrast grayscale images of stained nuclei **(C, G, K)** were generated to highlight changes in distribution between genotypes. Z-stack images were created looking onto the epithelial surface **(A, E, I)**, or onto the lens edge **(B, C, F, G, J, K)**. Plots of mean values of line scans (black lines, ± the SEM green bars) of EdU fluorescence taken across the lens diameter **(D, H, L)** showed that p110α/Cx50 dKO lenses had recovered peak proliferation in the equatorial germinative zone **(H)**, and that PTEN/Cx50 dKO lenses displayed EdU incorporation that peaked in the germinative zone **(L)**. Hoechst staining in the PTEN/Cx50 dKO lenses **(J, K)** illustrated that equatorial nuclei were diffusely distributed and displaced toward the posterior pole, compared to the dense band of nuclear staining seen in Cx50 KO and p110α/Cx50 dKO mice where new fiber cells become internalized (**B, C, F, G**, white arrowheads). Quantitation of central proliferation **(M–O)** revealed an elevated mean density of EdU positive lens epithelial cells in p110α/Cx50 dKO **(P)** mice compared to Cx50 KO or PTEN/Cx50 dKO animals (p < 0.05). n = 4-5 lenses per genotype. Bar = 0.5 mm. Open squares are raw data, filled circles are the mean ± SD. Asterisk indicates significantly different values compared to the Cx50 KO.

Single PTEN KO lenses were previously shown to display robust postnatal EdU labeling with a pattern similar to wild-type animals with the highest level of fluorescence in the germinative zone near the lens equator (68). By contrast, single Cx50 KO lenses previously showed decreased DNA replication on P2, especially within the central anterior epithelium (15). To examine mitosis on P2 in in p110α/Cx50 and PTEN/Cx50 dKO animals, lenses from Cx50 KO, p110α/Cx50 dKO and PTEN/Cx50 dKO mice were labeled with EdU. On postnatal day 2, Cx50 KO (**Figures 6A, B**), p110α/Cx50 dKO (**Figures 6E, F**), and PTEN/Cx50 dKO (**Figures 6I, J**) lenses all displayed the greatest levels of proliferation in the equatorial germinative zone. Similar to p110α KO mice (16), P2 p110α/Cx50 dKO lenses had recovered the deficit of proliferation in this region that was evident on P0. The mean values of line scans (black lines, ± the SEM green bars) of EdU fluorescence taken across the lens diameter confirmed that the p110α/Cx50 dKO lenses had recovered peak proliferation in the equatorial germinative zone on P2 (**Figure 6H**). All three of the Cx50 deficient lenses also showed decreased EdU incorporation in the central lens epithelium (**Figures 6M–O**), consistent with prior studies (15, 35), with the lowest levels observed in the Cx50 KO and PTEN/Cx50 dKO animals (**Figure 6P**). The Hoechst staining of nuclei in the PTEN/Cx50 dKO lenses (**Figures 6J, K**) further illustrated a loss of organization of nuclei in the equatorial bow region, compared with the dense band of nuclear staining seen in both Cx50 KO and p110α/Cx50 dKO mice where new fiber cells become internalized (**Figures 6B, C, F, G**, white arrowheads). Lens nuclei in the P2 PTEN/Cx50 dKO animals failed to internalize in the bow region and were diffusely distributed and displaced toward the posterior pole. These data suggest that the independent additive effects of transient loss of proliferating epithelial cells in the germinative zone on P0 and in the central zone on P2, as seen previously in the single p110α and Cx50 KO mice respectively could cause the significant reduction in lens size following p110α/Cx50 double deletion. They further show that double deletion of PTEN/Cx50 disrupted the spatial organization of lens nuclei.

### 3.7 Double deletion of PTEN/Cx50 resulted in histological abnormalities in the lens

**Figure 7:**
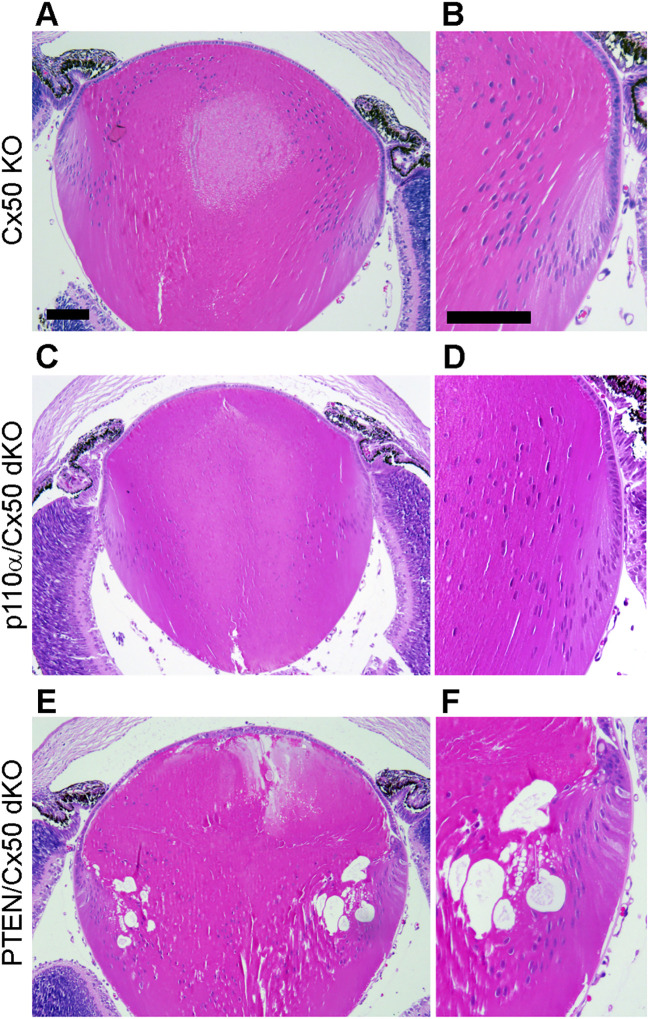
PTEN/Cx50 dKO lenses showed histological abnormalities. P2 eyes were fixed, embedded in paraffin and sectioned. Sagittal sections through the central region were stained with hematoxylin and eosin. Cx50 KO lenses **(A)** contained a central zone of reduced eosinophilic staining. The bow region of Cx50 KO lenses **(B)** showed delayed denucleation of differentiated fiber cells. p110α/Cx50 dKO lenses **(C, D)** were similar to Cx50 KOs, but had normal eosinophilic staining, and often displayed posterior defects. PTEN/Cx50 dKO lenses **(E, F)** had several histological anomalies. The bow region failed to form properly, with shortened lens fibers and their associated nuclei displaced toward the posterior lens. The equatorial area also contained large vacuoles, and additional histological defects were present in the anterior and posterior regions. Bars = 100 µm.

To compare the histology of Cx50 KO, p110α/Cx50 dKO and PTEN/Cx50 dKO lenses, eyes from mouse pups on P2 were dissected, fixed, sectioned and stained with hematoxylin and eosin. Sagittal sections through the central region of Cx50 KO lenses (**Figure 7A**) contained a central zone where the lens fibers displayed reduced eosinophilic staining as previously described (17). The bow region of Cx50 KO lenses was normal (**Figure 7B**), although there was evidence of delayed denucleation of differentiated fiber cells as formerly reported (13). Consistent with the absence of nuclear cataract shown in **Figure 1**, the p110α/Cx50 dKO lenses lacked the central zone of reduced eosinophilic staining (**Figures 7C, D**). In addition, p110α/Cx50 dKO lenses often displayed posterior defects that may represent early stages of lens rupture. By contrast, PTEN/Cx50 dKO lenses showed severe histological anomalies (**Figures 7E, F**). The bow region failed to form properly, with shortened lens fibers and their associated nuclei displaced toward the posterior lens, consistent with the Hoechst staining described above (**Figures 6J, K**). This equatorial area also contained large vacuoles, and additional histological defects were present in the anterior and posterior regions of PTEN/Cx50 dKO lenses. Thus, double deletion of PTEN/Cx50 from the lens induced several histological defects in the lens.

## 4 Discussion

We have generated and characterized p110α/Cx50 and PTEN/Cx50 double knockout mice with the goal of improving our understanding of the integration of gap junctional communication mediated by Cx50 with the intracellular signaling activity of PI3K and PTEN in the pathophysiology and normal physiology of the lens. As summarized in **Table 1**, double deletion of Cx50 and p110α resulted in a unique phenotype compared to both of the respective single knockouts. The microphthalmia, proliferation defects, and smaller sizes of single Cx50 KO and p110α KO lenses (15–17) were additively intensified in the p110α/Cx50 dKO. In addition, a novel rupture phenotype, never observed in the single KOs, appeared in the p110α/Cx50 dKO with a rapid time course of development. Double knockout of PTEN and Cx50 produced lenses with some features similar to each of the respective single knockouts. The smaller eye and lens size of the Cx50 KO was not notably altered by further removal of PTEN in the dKO. By contrast, the cataract and rupture phenotype seen in single PTEN KO lenses (60) was more severe and had a faster time course in the PTEN/Cx50 dKO mice. Two novel phenotypes also emerged, that were absent in the respective single KOs. PTEN/Cx50 dKO animals had postnatal lens fiber differentiation defects that resulted in disorganized and anteriorly displaced cell nuclei. Finally, a subset of PTEN/Cx50 dKO lenses displayed an anomalous association with blood vessels of the tunica vasculosa lentis during the first postnatal week.

The lens growth pattern in rodents undergoes a change at P0, the time point where we observed a large decrease in dividing epithelial cells in the germinative zone of p110α/Cx50 dKO lenses. During embryonic development, the diameter of the lens increases linearly while the lens volume grows exponentially (75, 76). During the first postnatal week, lens growth occurs in an oscillatory manner corresponding to epithelial cell cycle timing that can be simulated by pulsatile administration of growth factors to organ-cultured lenses (77, 78). In single p110α KOs, peak lens epithelial cell proliferation in the germinative zone vanished at P0, despite being clearly evident on E17 and P2 (16). In p110α/Cx50 dKO lenses, germinative zone peak proliferation also transiently vanished at P0, but returned by P2. This suggests that both embryonic and postnatal growth mechanisms can produce maximum cell division in the germinative zone that do not require the activity of p110α. However, the transition period between these two distinct growth mechanisms on P0 was affected by the absence of p110α activity, in a manner that was independent of the presence of Cx50.

Loss of Cx50 activity was also correlated with discrete temporal changes in epithelial cell proliferation linked to significant reductions in lens size. Knockout of Cx50, or its replacement with Cx46 by genetic knocking, resulted in significantly smaller lenses that failed to generate a single pulse of postnatal epithelial cell proliferation on P2 (15, 17, 23, 35). Cx50 functional activity reaches a peak in early postnatal epithelial cells when lens mitosis switches to the pulsatile growth pattern, and Cx50 conductance can be significantly upregulated by p110α activity (35, 61). The finding that the two distinct lens growth defects persisted independently in the p110α/Cx50 dKO, and resulted in a much smaller lens that either single KO, suggests that there was no functional epistasis between the Cx50 and p110α growth deficits, despite their ability to functionally interact *in vitro* (61).

The vast majority of p110α/Cx50 dKO lenses underwent a posterior rupture within 1 week of age. This was unexpected, as neither Cx50, nor p110α, single KO lenses ever displayed lens rupture (16, 17, 68). Both p110α and Cx50 play a central role in the regulation of intracellular hydrostatic pressure in the lens (62–64), so it is possible that the combined loss of their activities led to a catastrophic loss of homeostasis. PTEN antagonism of p110α signaling is critical to maintain lens homeostasis (68). In single PTEN KO lenses, unchecked p110α activity reduced epithelial cell Na^+^/K^+^-ATPase activity (60), causing reduced sodium and water efflux mediated by the lens circulation (79, 80), and resulted in increased intracellular hydrostatic pressure and lens rupture. The incidence and kinetics of lens rupture in the PTEN/Cx50 dKO was also accelerated compared to the single PTEN KO (60), highlighting the importance of Cx50 and PTEN in regulation of the lens circulation. Elucidation of the precise mechanisms whereby Cx50, PI3K and PTEN collaboratively preserve lens homeostasis will require additional investigation.

The PTEN/Cx50 dKO mice displayed two additional defects that were not previously observed in wild-type mice or the respective single KOs (13, 17, 60). First, blood vessels, presumably from the tunica vasculosa lentis, became highly adherent to a subset of PTEN/Cx50 dKO lenses between P2 and P7. The reasons for this alteration in tunica vasculosa structure, and how these changes were driven by the combined loss of Cx50 and PTEN in the lens are not known. Second, newly differentiated fiber cells in the bow region failed to elongate properly and migrated posteriorly, leading to displaced and disordered cell nuclei. The cytopathology in PTEN/Cx50 dKO animals was amplified when compared to the specific and limited histological defects present in either PTEN or Cx50 single KO lenses (17, 60). PTEN/Cx50 dKO lenses also showed a less organized band of new fiber nuclei in the bow region. Examination of the nuclear distribution by Hoechst staining showed that Cx50 single KOs had a clearly demarcated transition zone where the anterior epithelium ended and fiber differentiation began. By contrast, disordered surface nuclei persisted well toward the posterior pole in PTEN/Cx50 dKO lenses. Fiber cell differentiation is largely regulated by FGF signaling (5, 6, 81), although in the absence of PTEN the differentiation process can be influenced by non-FGF signaling pathways (7). Cx50 also affects lens fiber differentiation (13, 21, 82), and it is possible that the loss of both modes of communication could not be compensated for in the PTEN/Cx50 dKO lenses.

We have examined interaction between Cx50 mediated gap junctional communication and PI3K/PTEN signaling in the lens using an *in vivo* methodology involving mice with multiple gene knockouts. Double knockout of p110α and Cx50 significantly reduced eye and lens size, and resulted in a high rate of lens rupture starting in the first postnatal week. Cell proliferation defects identified in the single Cx50 and p110α knockouts were maintained in the p110α/Cx50 dKO lenses. Double deletion of Cx50 and PTEN produced severe lens defects, including cataract, aberrant cell migration, vacuole formation and lens rupture. Taken together, these observations suggest that interaction between PI3K/PTEN signaling and Cx50 mediated intercellular communication may participate in the regulation of lens cell proliferation, differentiation and homeostasis, and that loss of this regulation may contribute to a variety of developmental defects. Further study of how lens gap junction channels and PI3K/PTEN signaling work together to maintain clarity, preserve integrity and regulate postnatal mitosis could provide broad insights into the regulation of channel/transport activity in other tissues.

## Data Availability

The raw data supporting the conclusions of this article will be made available by the authors, without undue reservation.
